# Novel characteristics of soluble fibrin: hypercoagulability and acceleration of blood sedimentation rate mediated by its generation of erythrocyte-linked fibers

**DOI:** 10.1007/s00441-022-03599-9

**Published:** 2022-03-11

**Authors:** Dennis K. Galanakis, Anna Protopopova, Kao Li, Yingjie Yu, Tahmeena Ahmed, Lisa Senzel, Ryan Heslin, Mohamed Gouda, Jaseung Koo, John Weisel, Marilyn Manco-Johnson, Miriam Rafailovich

**Affiliations:** 1 Dept. of Pathology, Stony Brook University School of Medicine, Stony Brook, NY USA; 2 Dept. of Cell and Developmental Biology, Univ. of Pennsylvania School of Medicine, Philadelphia, PA USA; 3 Dept. of Materials Science and Engineering, Stony Brook University, Stony Brook, NY USA; 4 Dept. of Medicine, Stony Brook University School of Medicine, Stony Brook, NY USA; 5 Dept. of Physiology and Biophysics, Stony Brook University, Stony Brook, NY USA; 6 Heme/Onc and Bone Marrow Transplantation, Children’s Hospital, Univ. of Colorado, Aurora, CO USA

**Keywords:** Soluble fibrin, Erythrocyte sedimentation, Molecular imprints, Thromboelastography, Clot lysis, Atomic force microscopy

## Abstract

Soluble fibrin (SF) in blood consists of monomers lacking both fibrinopeptides A with a minor population in multimeric clusters. It is a substantial component of isolated fibrinogen (fg), which spontaneously self-assembles into protofibrils progressing to fibers at sub-physiologic temperatures, a process enhanced by adsorption to hydrophobic and some metal surfaces. Comparisons of SF-rich (FR) and SF-depleted (FD) fg isolates disclosed distinct molecular imprints of each via an adsorption/desorption procedure using gold surfaced silica microplates. Accelerated plasminogen activator-induced lysis and decreased stiffness (G′) of thrombin-induced FR fg clots were revealed by thomboelastography. Erythrocyte sedimentation (ESR) in afibrinogenemic plasma (Hematocrit 25–33%) was accelerated by FR fg nearly threefold that of FD fg. Stained smears disclosed frequent rouleaux formations and fibers linking stacked erythrocytes in contrast to no rouleaux by FD fg. Rouleaux formations were more pronounced at 4 °C than at ambient temperatures and at fiber-membrane contacts displayed irregular, knobby membrane contours. One of several FR fg isolates also displayed incomplete fiber networks in cell-free areas. What is more, pre-mixing FR fg with each of three monoclonal IgG anti-fg antibodies at 1.5 mol/mol fg, that inhibited fibrin polymerization, prevented rouleaux formation save occasional 2–4 erythrocyte aggregates. We conclude that spontaneously generated SF fibers bound to erythrocytes forming intercellular links culminating in rouleaux formation and ensuing ESR acceleration which in clinical settings reflects hypercoagulability. Also, the results can explain the reported fg binding to erythrocytes via ligands such as CD47, stable in vivo RBC aggregates in capillaries, and red areas of pathologic thrombi.

## Introduction

Soluble fibrin (SF) is substantially increased during withdrawal and storage of blood (Galanakis 1995) and is a component of isolated fibrinogen (fg). For example, when fg was isolated (Ware et al. 1947), SF most likely co-isolated with it and was later prepared and further investigated (Shainoff and Page 1962). A key attribute of SF is that at sub-physiologic temperatures and decreased ionic strength SF tends to form precipitates and gels that redissolve on rewarming to 37 °C (Shainoff and Page 1962; Shainoff and DiBello 2001; Galanakis et al. 2021). A possible early indication of the presence of SF emerged when fg precipitated from plasma, redissolved in NaCl solution (presumably normal saline), and coagulated on dilution with water (Denis 1859). Similar indications emerged in various reports well into the twentieth century showing low solubility of fg under sub-physiologic temperatures. Exploiting this, a group (Ware et al. 1947) showed that plasma frozen at −30 °C and thawed slowly displayed an insoluble component which after washing with ice cold saline dissolved by rewarming to 37 °C yielding pure fg. This led to thawing frozen plasma at 4 °C yielding a cryoprecipitate (Pool et al. 1964) which was rich in fg (in hindsight mostly SF), Factor VIII, von Willebrand Factor, Factor XIII, and it re-dissolved at 37 °C. This resulted in its extensive clinical use as replacement of these factors particularly Factor VIII for many years until respective factor concentrates became available. Plasma cryoprecipitate is still used today during massive transfusions and when the relative factor concentrates are not available. Using the cold washing observation (vide supra), we isolated SF by washing plasma cryoprecipitate with cold buffer (Galanakis et al. 2021). Another group (Morrison et al. 1948) purified fg by an ethanol and glycine precipitation method and their otherwise highly pure fg was heterogeneous by ultracentrifugation yielding an S_20_ value of 8.5 S with minor components showing 15 S and both higher and lower values. Similar heterogeneity (8 S and 24 S) reported later in studies of rabbit fg was attributed to dissociable fibrin/fg complexes, reviewed in Shainoff and DiBello (2001). In hindsight, this heterogeneity likely reflected the recently discovered multimeric SF/fg clusters (Galanakis et al. 2021).

In what recently proved to be SF-relevant investigations, increased sinking speed of RBCs resulting from their clumping was attributed to plasma (Nasse 1836) and this was co-present with increased fg concentration. The increased sinking observation led the Polish pathologist Edmund Biernacki in 1897 to devise the erythrocyte sedimentation rate test (ESR), the “Biernacki’s Reaction” (Grzybowski and Sak 2011) which in 1921 was standardized by Alf Vilhelm Albertsson Westergren and remains in use today as a clinical marker of inflammation (Westergren 1921; Harrison 2015; McCabe 1985). His contemporary Robin Fabraeus also used this test and thought that RBC aggregates occur in vivo (McCabe 1985). This view was corroborated by later reports showing RBC aggregates in retinal vessels in different clinical disorders (Glacet-Bernard et al. 1990; Harrison 2015). More recently, compelling correlations of ESR with carotid artery atheroma ((Singh et al. 2014) and with arterial and venous thrombosis complicating COVID-19 infection (Al-Samkari et al. 2020) were demonstrated, detailed in the "Discussion" section.

The second observation by Nasse, elevated fg levels in blood, has been consistently confirmed in inflammatory conditions in which fg is among 30 acute phase reacting proteins (Luyendyk et al. 2019; Davalos and Akassoglou 2012; Arbustini et al. 2013) including elevated fg during pregnancy (Hamilton 1953; Van den Broek et al. 2001). Over time, various clinical tests for SF were developed and its blood levels were also found elevated in inflammation by numerous reports. For example, elevated SF levels (also known as Cryofibrinogenemia) were found in 8–13% of hospitalized patients that included infection, malignancy, and autoimmune disorders such as severe cryoglobulinemic vasculitis (Michaud et al. 2015). Increased SF was also demonstrated in 12% of a large group of cryoglobulinemic patients (Saadoun et al. 2009). Meanwhile, the substantial SF levels shown in blood bank plasma (Galanakis 1995) raised the possibility of an SF role in the thrombotic complications of fg replacement therapy, particularly with use of the inherently SF-rich plasma cryoprecipitate, and multiple perioperative transfusions (Goel et al. 2018). Also, thrombotic complications within and around intravascular devices (Cost and Journeycake 2011) argue for a possible SF role, since many devices possess hydrophobic surface on which plasma SF readily adsorbs and self-assembles (Galanakis et al. 2021). Our present investigations of SF focus on its molecular imprint profile, stiffness and lysis of its thrombin-induced clots, and its possible role in ESR acceleration suggested by fg binding to RBCs (Maeda et al. 1984, 1987; De Olivera et al 2012; Lominadze and Dean 2002).

Fg is a 342-kDa major plasma glycoprotein which consists of three pairs of disulfide-linked non-identical polypeptide chains, Aα, Bβ, and γ. It is assembled in hepatocytes in two identical halves which are disulfide-linked at their central (or E) region where the amino termini of all six chains reside (Weisel 2005). Each of its two (outer or) D regions contains the carboxyl terminal end of each chain, and that of Aα chain, the longest, is tethered to the E region (Litvinov et al. 2007). This extended part is known as the αC region encompassing residues Aα221-610, which includes the αC connector, Aα221-391, and the αC domain, Aα392-610 (Medved and Weisel 2009). Thrombin cleaves two pairs of small fibrinopeptides, FpA and FpB, (Blomback and Yamashina 1958; Shainoff and Page 1962) from the N-termini of fg Aα and Bβ chains respectively on the central or E region (Weisel 2005). The cleavages expose pairs of “A” and “B” binding sites, known as “knobs” (α17-19 and β15-18, respectively) which interact with constitutively accessible complimentary “holes” or “pockets,” termed “a” (γ337-379) and “b” (β397-432) located and always accessible in each D region. The ensuing complimentary (knob A/pocket a) binding initiates the half staggered two molecule-thick fibril (protofibril) which also involves end to end DD contacts via γ375-409 (Everse et al. 1998), and when sufficiently elongated (Chernysh et al. 2011; Weisel and Litvinov 2017) assembles laterally with counterparts initiating generation of fibers and networks with varying fiber thickness and branching. Structures shown or hypothesized to contribute to interprotofibril binding, reviewed, and referenced by Weisel and Litvinov (2013) include knobs B and pockets b, C-terminal portion of the γ chains and two adjacent β nodules, αC regions, the coiled coils, and N-gycosaminoglycans at residues Bβ364Asn and γ52Asn. The fiber network is stabilized by thrombin-activated plasma transglutaminase (factor XIIIa or F XIIIa) that catalyzes formation of covalent γ-glutamyl-ε-lysyl intermolecular crosslinks (Smith et al. 2013). Four such links form on each α and two on each γ chain and they increase the clot resistance to proteolysis by plasmin.

## Materials and methods

### General

Reagents, supplies, and their sources were as detailed previously (Galanakis et al. 2021). Buffers used included the following: 0.01 M Tris–HCl, pH 7.4, in 0.15 NaCl (TBS); 0.01 M Na_2_PO_4_, pH 6.4 or 7, in 0.15 M NaCl (PBS); and 0.05 M or 0.02 M Na_2_PO_4_, pH 6.4. Line graphs and histograms were produced by use of the GraphPad Prizm program. Reptilase was obtained from Pentapharm (Basel, Switzerland), human thrombin from Enzyme Research Laboratories (South Bend, IN). D-phenyl-L-prolyl-L-arginine chloromethylketone (PPACK) and Dithiothreitol (DTT) were purchased from Calbiochem-Behring (La Jolla, CA), and Phenylmethanesulfonylfluoride (PMSF) from Sigma-Alrich (St. Louis, MO). For molecular imprinting, silica wafers, 0.5 × 2 cm, 3-mm-thick microplates with unpolished ~ 100-nm-thick gold surface layer, were purchased from the Cornell University Nanocenter (Ithaca, NY). Monoclonal IgG anti-fg antibodies (mAbs) were gifts from Dr. B. Kudryk and included T59, anti-γ385-406 that inhibited fibrin polymerization (Galanakis, unpublished clot turbidity time course data), and two others also shown to inhibit fibrin self-assembly (Koo et al. 2010), IC2-2, an anti-Aα529-539, and T103D, an anti-Aα241-476. Recombinant tissue plasminogen activator (rtPA) was a gift from Anthony B Chen. Plasma fg measurements were determined by the thrombin time (Clauss 1957) assay. For the Westergren ESR procedure, 1 ml pipettes were used. Erythrocytes, obtained fresh or within a week of phlebotomy, were washed × 3 in saline and resuspended in afibrinogenemic plasma to achieve the hematocrit specified in the results. Fg was added before or after the erythrocyte resuspension, mixed manually by gentle tapping, and placed in a slow rotating mixer for 5 min. To analyze RBC morphology, mixtures were allowed to stand for 30 min at ambient or 4 °C temperatures, resuspended, and smears were prepared on a glass slide. They were then subjected to Wright Giemsa staining, air drying, and examination by use of an optical microscope (Bain 2017).

### Fg and fibrin

Fg, >98% coagulable, was isolated by the glycine procedure (Mosesson and Sherry 1966) from human outdated plasma pools of 20 or more normal donors. Detailed in Galanakis et al. (2021), following their isolation SF-rich (FR fg) and SF-depleted (FD fg) fractions were dilipidated, dialyzed in 0.3 M NaCl, and stored in small aliquots in either polypropylene or polyethylene vials at −70 °C. As also described, although selected isolates were treated with 10 nM of either PMSF or PPACK for 30 min prior to freezing, no serine protease activity was detected by chromogenic assay of two untreated isolates. Prior to use, each thawed fg aliquot was diluted with an equal volume of deionized sterile water and further diluted in the desired buffer. In general, concentrations of FD and FR fg isolates tended to decrease up to 20% on thawing at 37 °C, a decrease attributable to adsorption to the vial surface**.** In addition, two of seven FR fg isolates formed minor amounts of gels which did not redissolve on gentle mixing and were synerized and removed. The following were used as described (Mosesson and Sherry 1966; Mosesson et al. 1974): spectrophotometric fg measurements, extinction coefficient of 15.5 (280 nm, 1 cm, 1%), electrophoresis in dodecyl sulfate polyacrylamide (SDS-PAGE), and isolation of des-αC fg. Estimated from the size of its Aα core remnants (Mosesson et al. 1974; Galanakis and Mosesson 1979), des-αC fg (old term fraction I-9), and from reported determinations of C-terminal residues (Asn-269, Gly-297, and Pro-309) of comparable preparations (Nakashima et al. 1992), such isolates lacked virtually all their αC-domain and variable C-terminal parts of their αC-connector. As described previously (Galanakis et al. 2021), Fg isolates were dialyzed ×3 at 4 °C vs 0.05 M Na_2_PO_4_ buffer, pH 6.4. Precipitates (FR fg) were harvested by centrifugation (4 °C), and supernatants (FD fg) were concentrated and were dialyzed in 0.3 M NaCl, stored at −70 °C, and thawed, 37 °C, prior to use. FR fg was also prepared by exposing plasma cryoprecipitate in situ to ice cold PBS buffer excess overnight ×3, dissolving in 0.3 M NaCl and storing frozen (vide supra). By SDS-PAGE, some (DDT-reduced) FR fg isolates displayed trace amounts of γ-γ dimers which were absent in fg isolated from cryoprecipitate-depleted plasma. SDS-PAGE electrophoretograms of the isolates were reported recently (Galanakis et al. 2021). Fibrin polymerization, monitored by turbidity (at 340 nm), was induced by thrombin 0.2 U/ml or Reptilase 1 U/ml, pH 6.4, and fg 1 mg/ml. The earliest time point of sustained rise in turbidity was used as clotting time.

### Molecular imprinting

This enables creation of binding sites with memory of the shape, size, and functional groups of the imprinted molecule (Yu et al. 2016). In the present application, a gold-surfaced silica wafer (vide supra) was immersed into a solution containing 100 μM 11-mercapto-1-endecanol, and 0.1 μg/ml fg, PBS**,** pH 7, for 60 min. The supernatant was then removed; the wafer was washed with 1 M NaCl to remove bound fg, and replaced with 100 ml phosphate buffer, pH 7, under slow stirring. Two separate electrode-linked plates were immersed in the buffer, one imprinted and one an Ag/AgCl plate, and each electrode-linked plate was linked to a potentiometer that yielded a linear voltage signal whose time course was recorded**.** Addition of a drop of fg, 100 μg/ml, resulted in a short lag followed by an abrupt decrease in voltage amplitude and a new baseline. Each additional fg drop added at intervals elicited a corresponding voltage change until additional drops elicited no further voltage change. The resulting negative voltage changes were plotted as a function of fg concentration yielding a sigmoid plot whose plateau reflected saturation or occupancy of all vacant imprints. Addition of a different protein (e.g., albumin or hemoglobin) elicited no voltage change and served as control.

### Thromboelastography

This was performed as described (Galanakis et al. 2014) using a model 5000 Thromboelastograph. As detailed in the results, rtPA was incorporated in a fg solution containing afibrinogenemic plasma, pH 7.4, as source of plasminogen just prior to adding thrombin. The maximum amplitude, MA, its decreasing slope, and point of no further change were used to compute the clot lysis rate and time by a thromboelastograph program. To compare MAs, FR and FD fg clots were prepared in the absence of platelets, which increase clot MA (Galanakis et al. 2014).

#### AFM

For imaging fg monomers and soluble polymers, adsorption to polystyrene (PS) and to trioctylmethylamine (TOMA) coated silica wafers was used and imaging was carried out by topographic and lateral atomic force microscopy (AFM) scanning as described (Koo et al. 2010; Galanakis et al. 2021). For enhanced image resolution, a modified graphite (MG) surface (water droplet angle 45.5° ± 0.5°, *n* = 3) was used as described (Protopopova et al. 2014) Briefly, a 2-μl aliquot of fg 5 μg/ml solution was applied for 5–15 s, immediately diluted with 100 μl deionized water for 10 s, dried by forced air, and scanned by AFM in tapping mode using super-sharpened cantilevers, SSS-SEIHR (Nanosensors, Germany) with a tip radius < 0.5 nm, and the MFP-3D AFM microscope (Asylum Research, Oxford Instruments, USA).

#### ESR

Sedimentation of erythrocytes (RBCs) in whole blood can be defined as initial rouleaux and other aggregate formation followed by sinking of the aggregates at a constant rate which eventually slows as aggregates pack at the bottom of the tube. We used the standardized gravity-dependent Westergren procedure, whereby pipettes with graduated markings are nearly filled with blood and immobilized in a vertical position. The top of the RBC column was measured at intervals, and the settling rate was expressed in mm/h.

### Functional Fg and fibrin structures

**Fig. 1:**
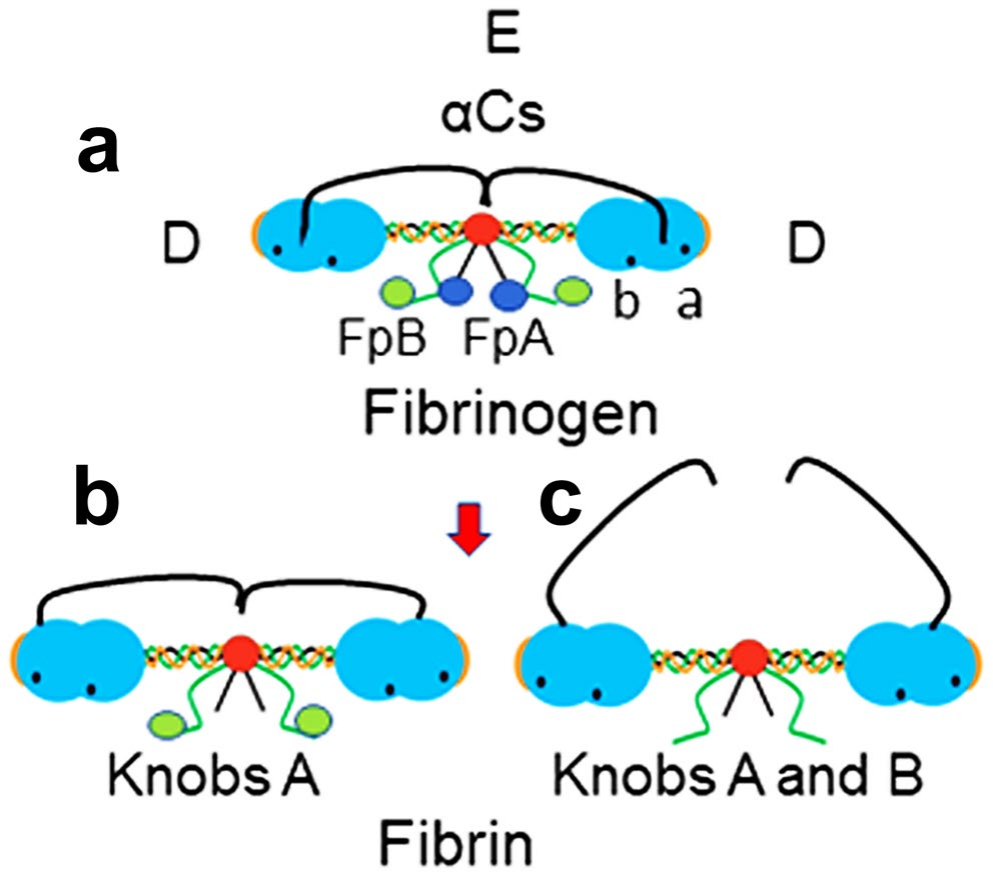
Molecular sites that participate in fibrin polymerization. **a** The cartoon illustrates the functional regions that participate in fibrin polymerization. The semi-lunar ends or red crests at the end of each D region reflect the peptide sequence, γ375-409 (Everse et al. 1998; Weisel and Litvinov 2013), engaged in DD contacts during elongation of protofibrils. The coiled coil connectors, top cartoon, between the D and E regions comprise interior parts of each chain and are thought to participate in contacts between protofibrils. Moreover, pockets “a” and “b,” top cartoon, are constitutively accessible on each D region of fibrinogen and fibrin and serve as structurally specific contacts of knobs A and B respectively. **b** This cartoon illustrates the absence of both FpAs, indicated on cartoon a, following cleavage by thrombin of each Aα Arg16-Gly17 bond (not shown). The resulting monomer initiates protofibril formation via intermolecular knob A/pocket a, αC/αC, and DD contacts. Evidence also suggests that the foregoing cleavage results in partial untethering of both αC regions from the E region, indicated in cartoon b. Such fibrin monomers comprise most of soluble fibrin in stored blood and that in circulation (Galanakis et al. 2021). **c** This cartoon illustrates the complete cleavage of both peptide pairs, FpA and FpB, and untethering of both αCs. Among characteristics of FpB cleavage per se is that it is slower than that of FpA, occurs mainly after protofibril is initiated, and enhances lateral protofibril/protofibril contacts. Such contacts are also enhanced by αC/αC contacts. What is more, recent evidence (Galanakis et al. 2021) strongly suggested that both protofibril/fg clusters and des-AA monomers participate in elongation and that the untethering of αCs in des-AA monomer is sufficient to promote fiber generation by lateral inter-protofibril contacts. Other evidence, moreover, Koo et al. (2010) implied that structures in the connector part of αC (Aα 221–371) promote coarse fiber generation, while others in the αC domain part (Aα 392–610) promote fine fiber generation

Figure 1 cartoon illustrates the molecular sites of fg and fibrin that enable fibrin self-assembly. While the “a” and “b” pockets are constitutively present, exposure of knobs A and B follow thrombin release of FpA and FpB. Knob/pocket interactions per se typically take place in liquid phase but can also occur when SF is adsorbed on hydrophobic or metal surfaces (Koo et al. 2010, 2012), precipitated by protamine (Stewart and Niewiarowski 1969), or subjected to cryoprecipitation conditions (Galanakis et al. 2021). During protofibril elongation, there is also intermolecular D/D contact at sites indicated on the outer part of the D shown as a red crescent in the cartoons. Untethering of αCs results from FpA and FpB release as shown, panels b and c. As detailed in the legend, this exposes sites on the αC connector and domain which promote fibril and fiber assembly the latter favoring lateral contacts resulting in fine and coarse fibers and the former elongated protofibrils (Koo et al. 2010).

## Results

### Molecular imprint comparisons

**Fig. 2:**
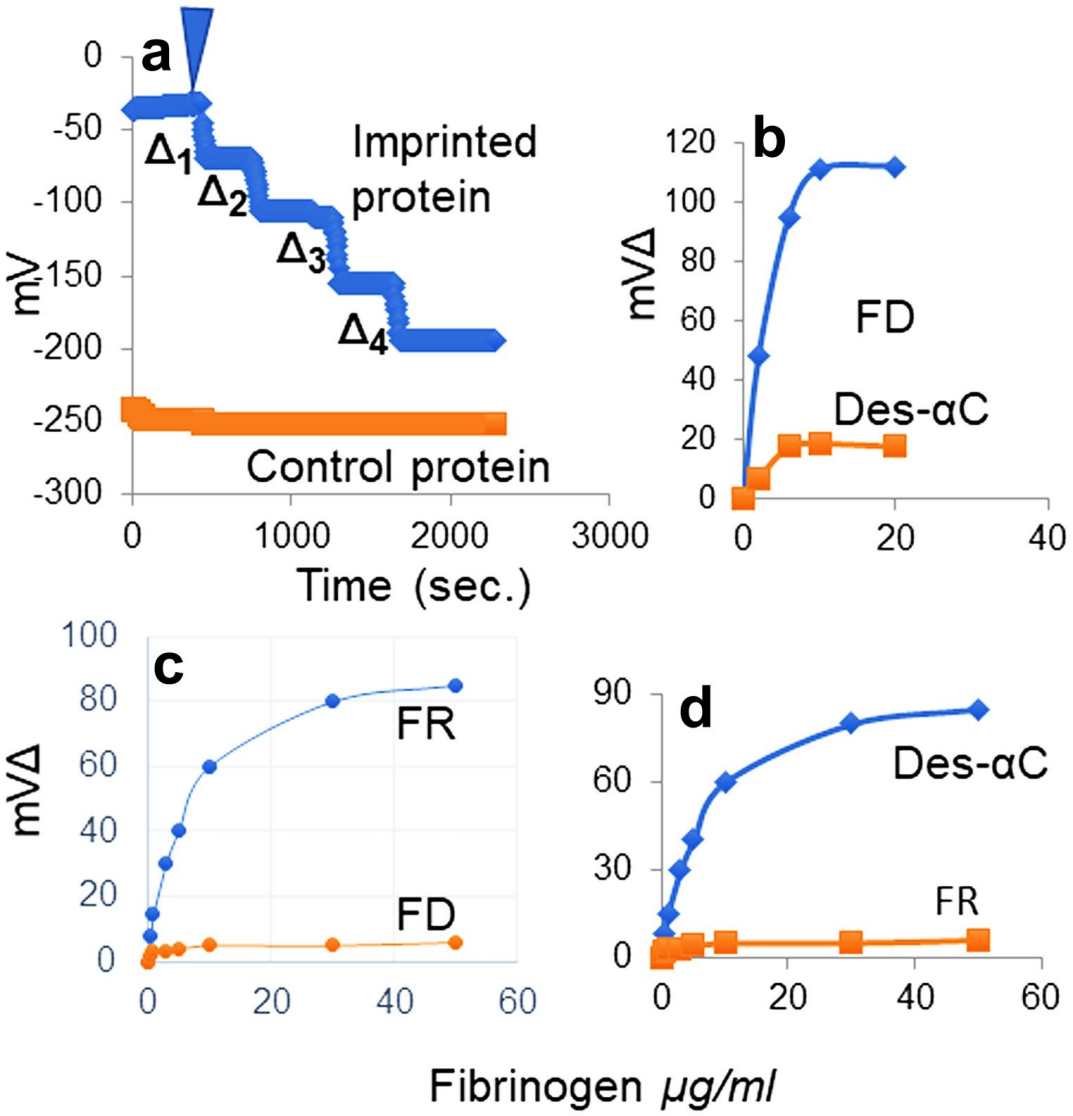
Demonstration of Molecular Imprinting Profiles. **a** This panel shows the experimental procedure whereby a step wise decrease in voltage follows incremental re-introduction of the imprinted protein. The voltage decrease shown as Δ followed the first drop of the imprinted protein solution, arrowhead, which is in turn followed by a new plateau. A second drop repeated this response, and it continued until new drops no longer elicited the response. The final plateau implied that all molecular cavities or imprints were occupied. The superimposed bottom graph is that of a non-imprinted or control protein showing no response to an identical plate. **b** The voltage Δ (illustrated in panel **a**) is graphed as a function of fg concentration shown from a single experiment using FD fg to imprint and an identically imprinted plate showing negligible or no responses to the αC fg control. **c** Graph of responses by FR fg re-introduced to its imprinted plate also showing no responses by an identical plate to FD fg, bottom graph. d Graph of from an experiment using plates imprinted with des-αC fg and FR fg as control

A variety of disciplines have used imprinting to distinguish molecules or particles from their otherwise similar counterparts since the 1930s. Imprinting profiles are determined by molecular configuration, size, charge, shape, and related differences among otherwise similar particles or molecules. In our adaptation of the procedure (Yu et al. 2016), detailed in “Materials and methods,” we used a gold surfaced microplate that was exposed to a mixture of fg and 11-mercapto-1-endecanol in buffer. The resulting surface coating comprised a covalently linked endecanol monolayer with randomly and non-covalently adsorbed fg. Following its desorption by 1 M NaCl, fg was re-introduced under potentiometric conditions in PBS, pH 7, eliciting a fg concentration-dependent negative voltage change **(**Fig. 2a). Using this procedure, we reported that the molecular imprint profile of the carcinoembryonic protein (found in tissues of the developing embryo and increased in certain post-natal or adult malignancies) was unique to this protein (Yu et al. 2016). Here we reasoned that since SF consists mostly of des-AA monomers its molecular imprints may be distinct from their fg counterparts and serve as a marker of their presence. Plotting voltage responses as a function of fg concentration, yielded a sigmoid plot with a steep rise ending in a plateau every time. The plot could not be elicited by a structurally different fraction exposed to an identical microplate and this served as control (Fig. 2b–d). That is, each plot pair (test and control sample shown) represents results of separate experiments whose plots are superimposed for clarity. As shown, they revealed that the plot was a distinct marker of the imprinted fg fraction. Such distinct plots were demonstrated by FD fg (Fig. 2b), FR fg (Fig. 2c), des-αC fg (Fig. 2d), and whole or parent (unfractionated), not shown. Also not shown, three genetic fg variants with single amino acid substitutions at different loci displayed no or negligible imprinting differences. Consequently, the distinct plots of FR and FD fg shown likely reflected partly untethered αCs, the presence or absence of FpA, or both, and the des-αC fg plot (Fig. 2d), reflected the absence of its αC region.

### Plasma fg measurements

**Fig. 3:**
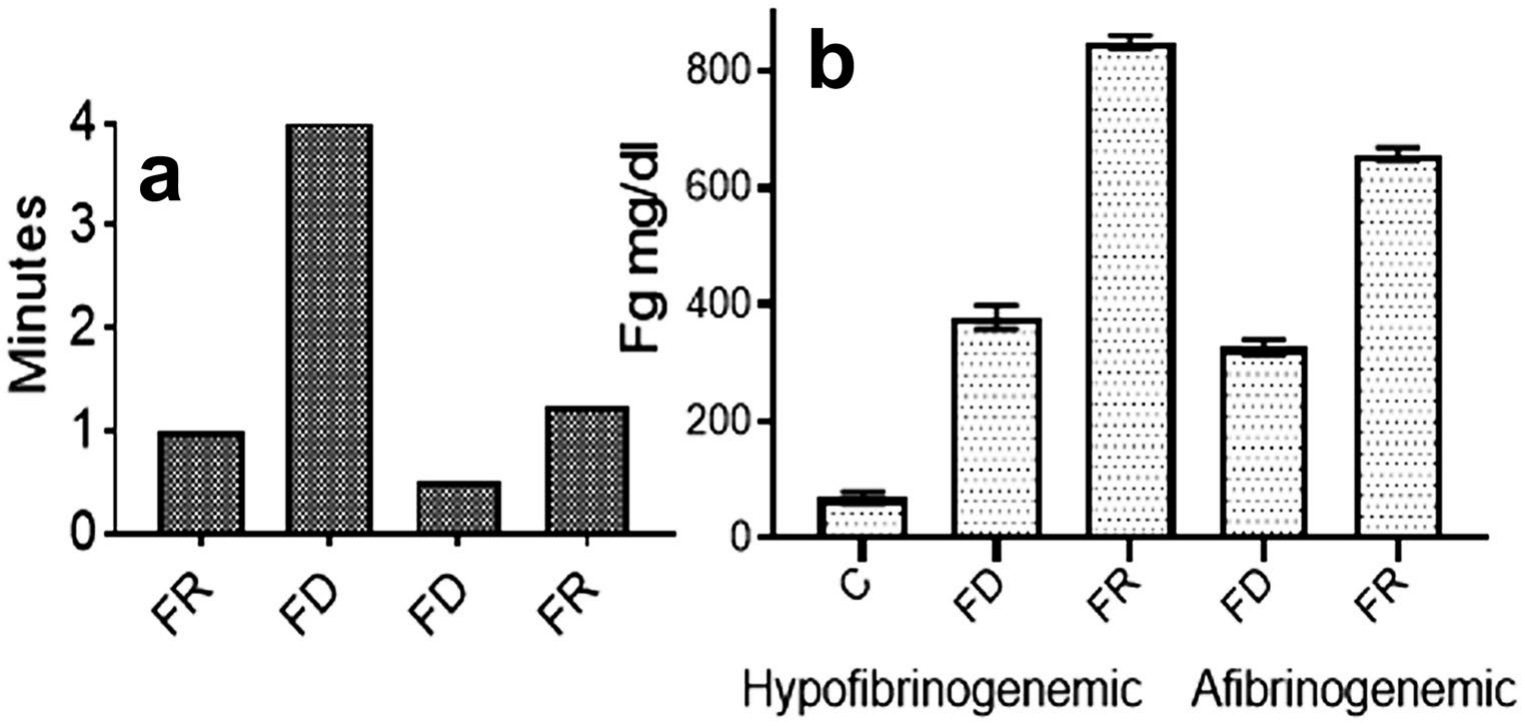
Clotting times and plasma Fg levels. **a** The accelerated thrombin clotting times, shown as the average of two measurements**,** also reported previously (Galanakis et al. 2021) and led to plasma fg measurements. **b** Plasma fg was measured by the Clauss (thrombin time) procedure. Shown are levels approximately twice those predicted by the addition of FD fg in both hypofibrinogenemic**,** native fg 67 mg/dl or C, (left two columns), and afibrinogenemic plasma (right two columns). To each milliliter of test plasma, 3 mg FD or FR fg was added, mixed, and allowed to stand 30 min. before testing. Each column indicates the average and range of duplicate measurements on the same plasma aliquot. The longer Reptilase clotting times, right column pair of panel a, were also characteristic of FR unrelated to the plasma fg measurements, and further considered in the "Results" section

Induced by thrombin, SF clotting times were shorter than controls (Galanakis et al. 2021) and were repeated here (Fig. 3a), left two columns, led to the question of their possible effect on plasma fg measurements. To be sure, fg levels based on a prothrombin time (PT) assay are commonly used but the thrombin time (Clauss) assay is also used for selected clinical specimens (e.g., hypofibrinogenemia and dysfibrinogenemia). We measured plasma fg concentrations following addition of FR or FD fg, and the PT procedure yielded no consistent differences between the two isolates. By contrast, measurements using the Clauss assay disclosed an approximately two-fold higher fg level resulting from the FR fg addition relative to that of FD fg (Fig. 3b). While this serves to characterize FR fg, the results cannot be extrapolated to clinical examples since such specimens they were not used here. An additional characteristic of FR fg consisted of prolonged Reptilase  clotting times relative to those of FD fg (Fig. 3a), right two columns which we attributed to the SF content of FR fg. That is, they reflected a significantly decreased fg population with intact FpA. By extension, the short thrombin clotting times of FR fg indicated a striking enhancement of polymerization attributable to exposure of its knobs B (i.e., Reptilase does not cleave FpB).

### Thromboelastographic measurements

**Fig. 4:**
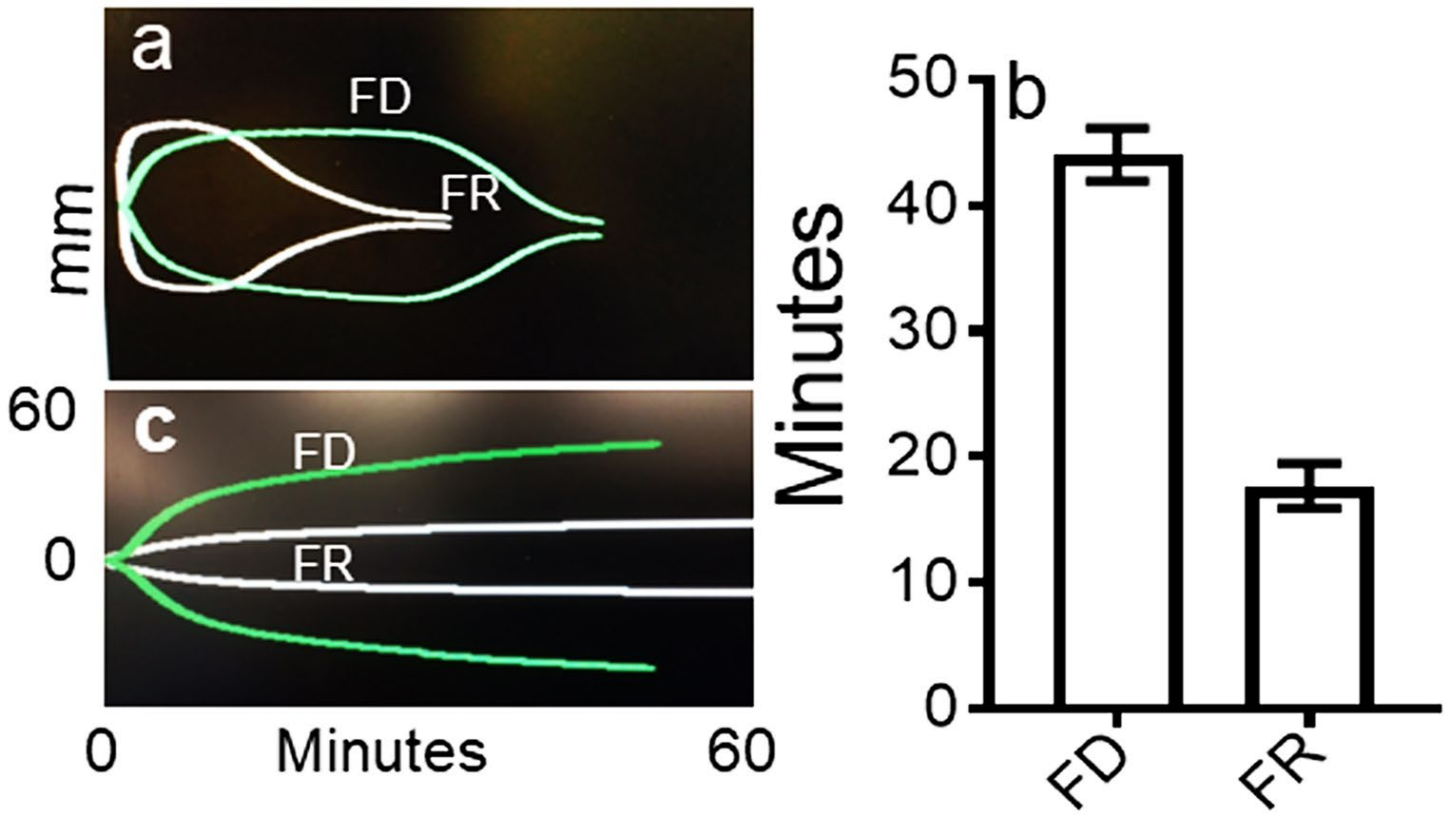
Thromboelastographic results. **a** Superimposed graphs of FR and FD lysis time course of single thrombin clots. To a solution of 3 mg/ml fg in 15% afibrinogenemic plasma containing 70 nM rtPA, pH 7.4, and platelets (170 × 10^3^/μl), thrombin was added to 0.4 U/ml final concentration. Computed lysis time of the FR clot from the graph shown was 12.67 min. and that of the FD clot 37.75 min. **b** The panel shows a similar difference in the range of lysis times of three other clots of each. Not shown were the clot lysis rates computed from the slopes in mm/min. which were 3.27 and 3.07 by single FR and FD clots, respectively.** c** Superimposed graphs of single clots showing decreased MA by the FR clot. These clots were obtained as in panel a without rtPA or platelets. MA of 11.8 and 43 mm of FD and FR clots respectively were used to compute corresponding Pas mentioned in the Results section

Evidence detailed in our recent report (Galanakis et al. 2021) suggested that areas in thrombin-induced fibrin clots comprise distinctly finer and non-branching networks with fiber-linked multimeric clusters that alter physical and functional clot properties, in general agreement with another report (Garcia et al. 2020). To explore this further two sets of experiments were carried out. In one set, clot lysis was induced by rtPA in afibrinogenemic plasma as a source of plasminogen (Fig. 4 a and b). To maximize MA, the clotting mixture contained platelets (Galanakis et al. 2014). These disclosed clearly faster lysis times of FR than of FD clots consistent with exposure of plasminogen and tPA binding sites further detailed in the “Discussion” section. In the other set, specified in the Fig. 4 legend, clot MAs were compared and disclosed decreased MA (Fig. 4c), by the FR fg clots indicating decreased clot stiffness (i.e. decreased elastic modulus or G′). Computed from MA values indicated in the legend of Fig. 4, G′ of FR clots was 68.2 Pa compared to 377 Pa of the FD clots, in general agreement with our G′ measurements obtained by a rotational rheometer (Galanakis et al. 2021). These results indicated that apart from the acceleration of thrombin-induced polymerization, FR fg formed clots with markedly decreased elastic modulus.

### Imaging by AFM

**Fig. 5:**
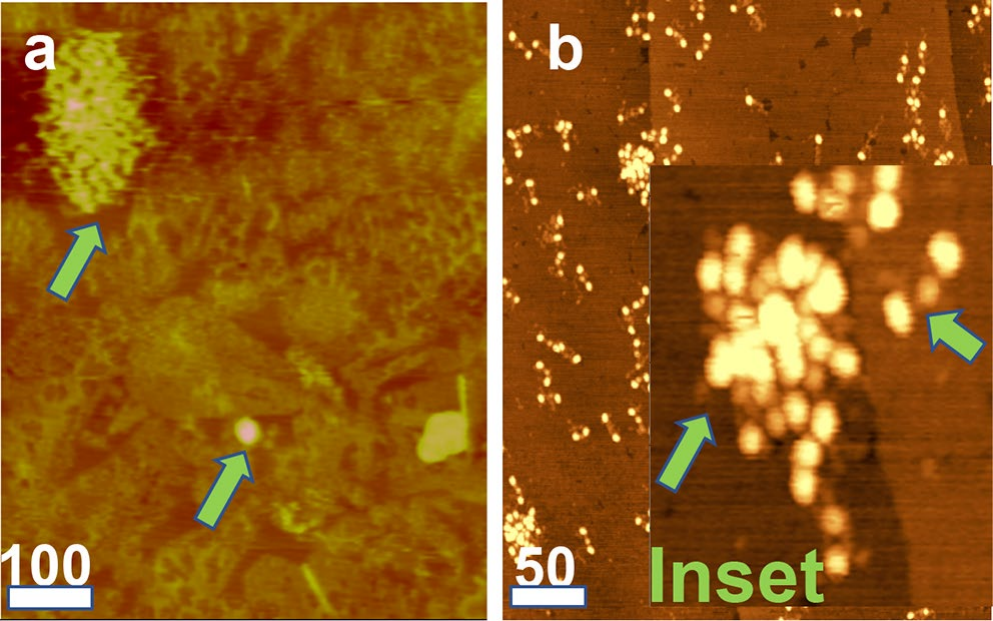
AFM images. Horizontal bars denote 100 nm and 50 nm in panels a and b, respectively. **a** FR fg adsorbed on a TOMA surface showing a large, upper arrow, and a smaller multimeric cluster, lower arrow, showing the marked variation in the cluster size. The smaller cluster also shows regular surface undulations indicating its multimeric composition. **b** FR fg adsorbed on modified graphite (MG) surface, showing a field of solitary trinocular monomers and two clusters. Inset: a 4 × magnification from a different area of the same field showing a monomer, right arrow, and a multimeric cluster, left arrow

The next set of AFM images mirror those in our previous report (Galanakis et al. 2021) and are presented here owing to their potential importance in the clot lysis and ESR experiments. One shows FR fg clusters adsorbed on the hydrophobic TOMA surface (Fig. 5a), that include a large cluster, left arrow, and smaller one with undulating margins, right arrow. In Fig. 5b, two small FR fg clusters are shown among numerous monomers. The inset is a 4 × magnification relative to Fig. 5b, and shows a cluster, left arrow, and monomer, right arrow, with its distinct E and D regions.

#### ESR

**Fig. 6:**
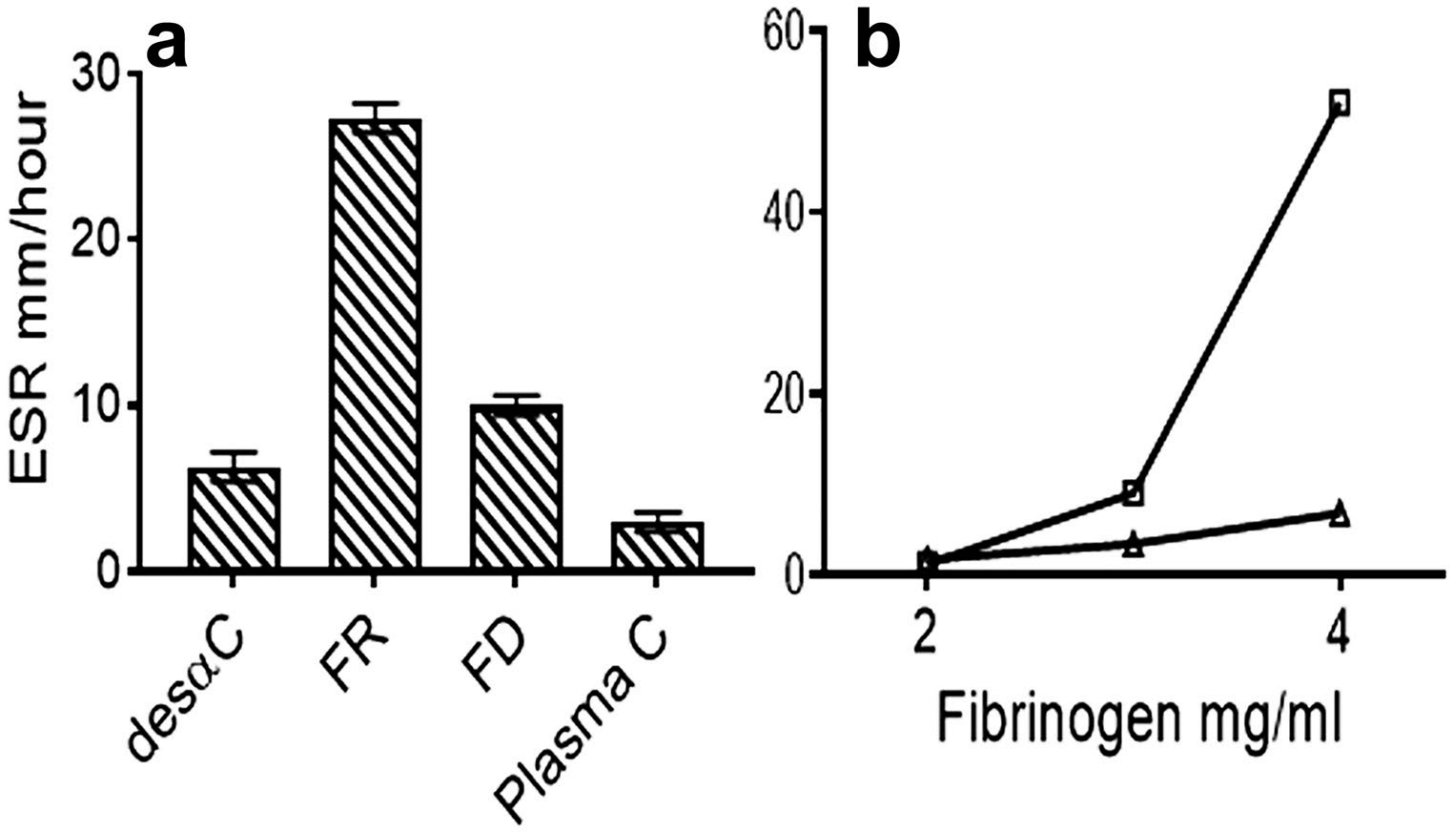
Accelerated ESR by FR fg. **a** Columns showing duplicate ESR measurements, range in brackets, obtained by the Westergren procedure, hematocrit 30%, fg 3 mg/ml, and 30% afibrinogenemic plasma, in TBS. They indicate a marked rate increase by FR as compared to those of its FD counterpart and those of des-αC fg. Plasma C: afibrinogenemic plasma controls with no added fg. **b** ESR effects by FR (squares) and FD (triangles) fg at three different fg concentrations obtained in 0.7% defatted human albumin in TBS and no plasma, hematocrit as in panel **a**. Each plotted point is from a separate experiment

Accelerated ESR has long been regarded as an indicator of inflammation (Luyendik et al. 2019) and of pregnancy (Van den Broek and Letsky 2001). Moreover, numerous studies identified fg as the major ESR enhancing protein, reviewed in Brust et al. (2014) who also described fg-dependent RBC microaggregates in capillaries. Such reports led to investigations of possible fg binding to RBCs. One group (Guedes et al. 2018) used a recombinant γ′γ′ fg, a homodimeric normal variant with an extended C-terminal sequence (Wolfenstein-Todel and Mosesson 1980) and described increased binding force and frequency of binding to RBCs as compared to recombinant γAγA fg, the common circulating form. Although this variant is present in < 5% of plasma fg, it may enhance fiber binding to RBCs. In another report (De Olivera et al. 2012), young RBCs were shown to be more active in binding fg than their senescent counterparts, and binding was blocked by mAbs against CD47 suggesting this as a fg integrin. In yet another report (Maeda et al. 1984), fg binding was tighter when RBCs were desialylated, thus discounting sialic acid as a fg binding ligand. Of interest also, fg bound to RBCs with a kd of 1.3 μM and binding was blocked by the RGDS peptide (Lominadze and Dean 2002). A related report (Maeda et al. 1987) showed decreased ESR by fg fragments X and Y both lacking intact αCs and thereby their RGD (Aα 572–574) motif. Since this site is exposed in fg (Peerschke and Galanakis 1987), we reasoned that it may play a role in SF binding to RBCs. Consequently, we included des-αC fg that lacks both αC regions containing this site in our ESR experiments. To explore the possible role of SF in ESR, advantage was taken of its inherent capacity to polymerize slowly at ambient temperatures, an attribute more marked at refrigeration temperatures. In one of three sets of experiments, washed RBCs were resuspended in buffer containing citrated afibrinogenemic plasma to achieve hematocrits ranging 29 to 33%. Fg was added to 3 mg/ml, gently mixed, and the ESR of the mixture was monitored (see “Materials and methods”). In an initial set of duplicate experiments, not shown, FR fg at 1 mg/ml displayed a baseline ESR of 7 and 9 mm/h which was near that of the afibrinogenemic plasma control. In sharp contrast, at 4 mg/ml acceleration increased to 47 and 49 mm/h. In further experiments (Fig. 6a), a modest acceleration by FD fg and by des-αC fg (undepleted of its SF) was evident relative to that of plasma controls. By comparison, acceleration was nearly tripled by FR fg as shown. In the third set, the dose response of acceleration was also shown in a 0.7% albumin solution without plasma (Fig. 6b).

**Fig. 7:**
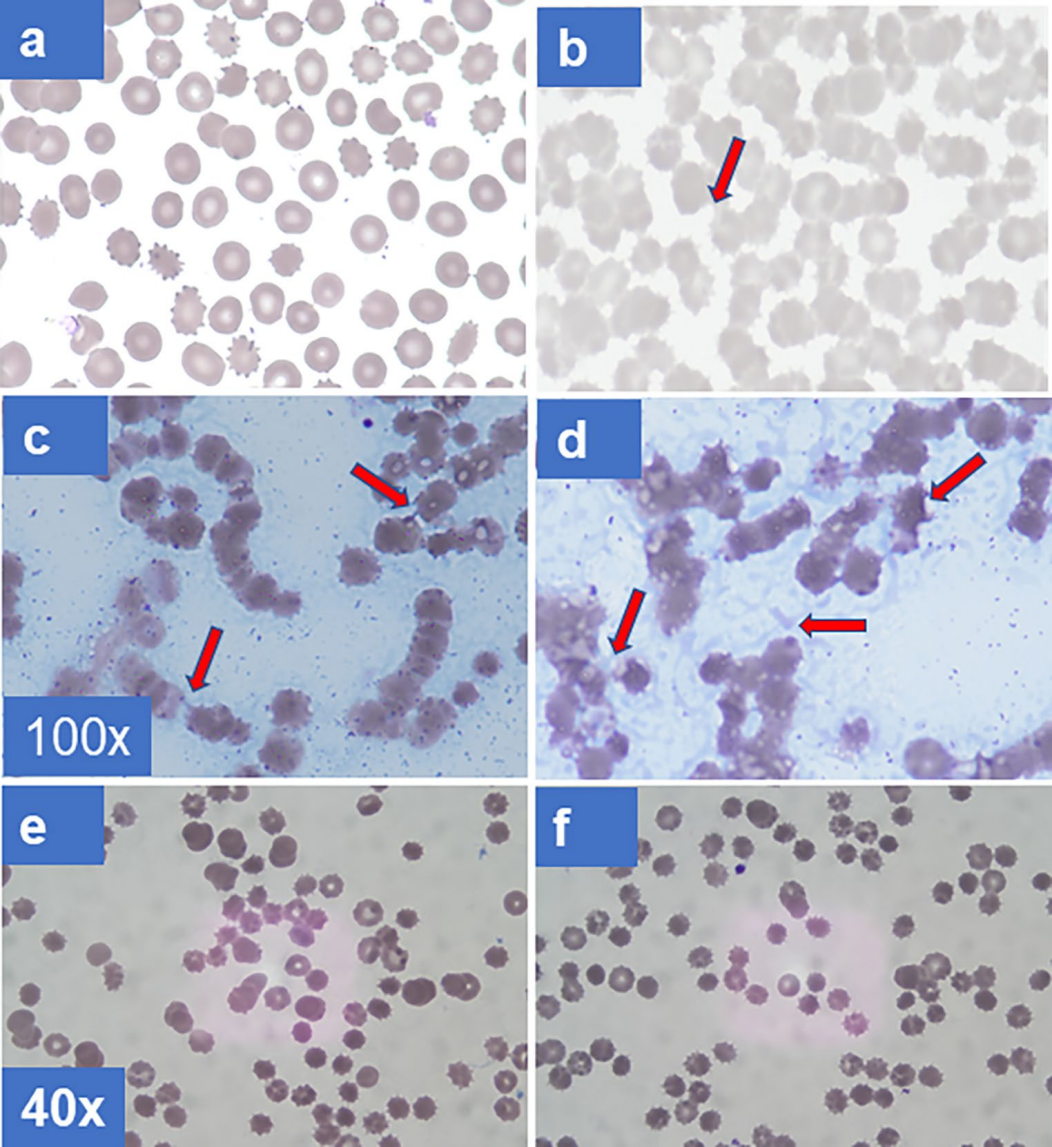
Rouleaux formation of RBCs by FR fg at ambient temperature. 100 × for panels **a**–**d** and 40 × for panels **e**–**f** indicate the objective lens size used to obtain the microscopic views shown. **a** Wright**-**Giemsa-stained glass slide smear of washed RBCs from a normal group O donor, hematocrit 30%, after incubation for 30 min. with afibrinogenemic plasma 30%, and FD fg 3 mg/ml. RBCs used were from a donor specimen kept refrigerated for 5 days and washed with excess 0.15 M NaCl before just before the experiments. **b** A separate experiment using conditions as in panel **a** and RBCs from the same specimen but incubated with FR fg showing frequent short rouleaux formations and many links between RBCs, one indicated the arrow. Two other pairs of experiments, not shown, yielded similar results. **c** A single experiment showing RBCs in more marked rouleau formations. FR fg + RBCs were mixed as in panel **a** but incubation was extended for 10 min at 4 °C before preparing the smear. Note that virtually all RBCs are in rouleaux formations and there are many links between RBCs in the rouleaux stacks, two indicated by arrows. **d** A single experiment with another FR fg isolate also showing most RBCs in rouleaux formations. It additionally shows poorly organized short fibers in most of the cell free background, horizontal arrow, and there are many links between RBCs. The left arrow indicates such a link and fibers overlying RBCs. The right arrow indicates deformations on an RBC surface that appear linked to fibers. Other RBCs show knobby membrane contours also linked to fibers. Results shown in panels **e** and **f** are from pre-incubation of FR fg with each of two IgG anti-fg mAbs. Panel **e** shows results from pre-incuabation with the anti-Aα529-539 mAb, and panel **f **from pre-incubation with the anti-γ385-406 mAb. Similar results, not shown, were obtained by pre-incubation with the anti-Aα261-476 mAb. Although occasional fiber links between two to four RBCs in linear formation are still evident in both panels, these RBCs are not closely packed or in rouleaux formations. RBCs for the experiments were from another group O donor specimen refrigerated for several days before the experiments

These results led to examination of the possible effect of FR fg on rouleaux formation which is regarded as central to ESR acceleration (Yin et al. 2017). Accordingly, combined in TBS were mixtures of 30% RBCs v/v, 30% afibrinogenemic plasma, and fg 3 mg/ml. They were gently mixed, allowed to stand for 30 min, and tapped to re-suspend, and their glass smears were stained and examined. The results disclosed rare RBC doublets by FD fg (Fig. 7a), in contrast to frequent rouleaux formations by FR fg (Fig. 7b). The smears showing rouleaux aggregates also revealed frequent links between RBCs, one shown by the arrow in (Fig. 7b). This was attributed to the characteristic tendency of FR fg isolates to form insoluble fibers at ambient and sub-ambient temperatures particularly on diluting their solutions from 0.3 M NaCl to 0.15 M needed for the experiments. Stained smears of mixtures using two different FR fg isolates exposed to 4 °C (30 min.) disclosed clearly more marked rouleaux formation (Fig. 7 c and d), also confirming the presence of fibers between RBCs, arrows. Additionally, one FR fg isolate displayed fibers in the cell-free background which were not organized into networks, one area shown by the horizontal arrow in (Fig. 7d). Moreover, there were as many as 2 to 3 fibers linking two RBCs, and they appeared of similar thickness to those in the cell-free background. Fibers also appeared to surround rouleaux formations, left arrow in Fig. 7d, and some RBCs displayed an acanthoid-like or knobby appearance, right arrow. What is more, RBC aggregates in the left lower and upper fields of Fig. 7d displayed thick fibers overlying RBCs in continuity with those linking RBCs, arrow in left lower corner of panel d. To further assess the role of fibers in rouleaux generation, three mAbs shown to inhibit fibrin polymerization (see “Materials and methods”) were incubated (10 min.) with FR fg before each experiment at a molar fg: mAb ratio of 1:1.5 and ambient temperature before adding RBCs and subjecting mixtures to 4 °C for 30 min. All three mAbs prevented rouleaux formation save a few fibers linking 2–4 RBCs (Fig. 7e–f). The results established that fibrin fibers were critical to rouleaux generation and by extension to ESR acceleration.

## Discussion

The most important novel characteristic of SF was its marked acceleration of ESR, revealing that the long-known temporal association of the acceleration with elevated fg levels reflected a direct effect by its SF component. Key to this effect was the inherent tendency of SF to spontaneously polymerize during slow thawing at 37 °C and 0.3 M or lower NaCl concentrations and temperatures. SF-depleted isolates (FD fg), by contrast, were not cryoprecipitable and did not display fiber generation (Galanakis et al. 2021). The stained smears revealed frequent fiber links between RBCs in the rouleaux stacks implying they mediated stack formation but the mechanism remains unclear. It is possible that either higher link density or proximity of peripheral surfaces or both favored binding and stable stacking assembly of the discoid RBCs. If so, this can explain why spherocytes typically do not form rouleaux (Tishkowski and Gupta 2021). Also mentioned in that and other reports the electronegative charges of RBC membranes are believed overcome by positively charged proteins. Our data shown in Fig. 7b–d strongly suggest that this electronegative zone, generally thought to be around 250 nm wide, is easily bridged by the length of fibrin fibers linking one cell to another. Supporting the conclusion of a central role by SF in rouleau formation were experiments revealing that fiber contact sites on RBCs displayed membrane distortions including irregularly spaced knobs and spicules (Fig. 7d), suggesting tight binding. Moreover, firm evidence for this conclusion was provided by their virtual prevention when fibrin polymerization was inhibited (vide supra).

RBC structures that accounted for the putative fiber-RBC links remain largely undetermined. Two reports suggested CD47 (Maeda et al. 1987) and unidentified RGD binding site(s) (Lominadze and Davis 2002), but these remain to be further defined. Moreover, RBC aggregation and/or rouleaux generation commonly attributed to increased immunoglobulins in paraproteinemias raises the possibility of ligands specific for them notwithstanding a role by accompanying SF that has not been explored. Interestingly, albumin retarded ESR in plasma (Reinhart and Dean 1995), in contrast to its acceleration in a buffer mixture of fg and immunoglobulins. These effects are consistent with the synergistic inhibition of fibrin polymerization by fg and albumin in molar excess (Galanakis et al. 1987). However, albumin in molar excess alone exerted acceleration of polymerization, an effect attributable to its hydrophilic and thereby water exclusion capacity. This is consistent with the present demonstration (Fig. 6b) of concentration-dependent ESR acceleration by FR fg in 0.7% albumin in buffer. What is more, failure of des-αC fg to increase the ESR rate reflected its lower (5–8%) SF content, relative to that (e.g., > 30%) of intact FR fg apart from possible participation by the fibrin αC region that such isolates inherently lacked (Galanakis et al. 2021).

Among other FR fg characteristics of FR fg (i.e. SF) were its prolonged Reptilase clotting time. This implied that polymerization by cleavage of FpA was not enhanced by the pre-existing population of des-AA fg. By contrast, its markedly short thrombin times indicated enhanced polymerization by pre-exposed knobs A in combination with a likely similar effect by newly exposed knobs B. This SF capacity clearly implies for the first time that in a thrombin generating environment, it polymerizes far more rapidly than fg. By extension, abnormally elevated SF levels reflect a risk for or ongoing pathologic thrombosis. Another SF characteristic was the decreased G′ of thrombin induced clots (Fig. 4c), intimating abnormal clot structure as recently reported (Garcia et al. 2020; Galanakis et al. 2021). Also typical was its rapid clot lysis (vide supra) attributable its altered clot ultrastructure and to exposure on fibrin of two critical clot lysis promoting sites. These comprise α148-160 that binds plasminogen and γ312-324 that binds plasminogen activator (Yakovlev et al. 2000; Medved and Nieuwenhuizen 2003) which are cryptic on fg. During fibrin self-assembly, D-E interactions result in conformational changes of the D region that expose these sites while another cryptic set of plasminogen and tPA binding sites in the compact region of αC (392–610) are also exposed. What is more, all these are necessarily exposed on the protofibril core of SF clusters which were either incorporated in or closely linked to network fibers (Galanakis et al. 2021). Consequently, the preformed cluster core initiates plasmin generation apart from that by the fiber network per se, and this potentiating effect can explain the more rapid lysis of FR than of the FD clots (i.e. FD is largely devoid of SF and its clusters). A different unique FR fg characteristic comprised distinct molecular imprints which were attributable to its untethered αCs, exposed knobs A, or both and served as a distinguishing marker of FR fg. The distinct imprint profile of des-αC fg is clearly attributable to its lack of the intact αC region.

### Clinical implications

According to laboratory manuals **b**lood specimens submitted for the ESR (Westergren) test must by standard procedure be tested within 2 h of storage at ambient temperature or several times longer in refrigeration. These conditions can easily result in fibers that bind to RBCs. Two recent clinical studies lent support to our conclusion on the SF role in ESR. In one, accelerated ESR was shown within 12 h of ischemic carotid artery stroke (Singh et al. 2014) with a clear correlation with atheroscrelosis markers in 96 patients, *r* = 88, *P* < 0.0001, and with the presence of carotid artery plaques *P* < 0.026. In the other, a study of 400 hospitalized patients with COVID-19 infection (Al-Samkari et al. 2020), thrombotic complications other than DIC which was rare, occurred in 9.5% (including arterial thrombosis in 2.8%, and venous thromboembolism in 4.5%). Among co-present thrombosis markers were elevated fg and accelerated ESR, and markers predictive of complicating thrombosis included elevated platelet count, CRP, DD-dimer, and ESR. Additionally, evidence of modestly increased fibrin monomer complexes (FMC) in pregnancy (Hafter et al. 1975; Onishi et al. 2007) likely reflected the multimeric clusters we recently described (vide supra). Since the clusters comprise a minor SF population, their elevated levels represented more substantial SF elevations and could thereby explain the long known marked acceleration of ESR in pregnancy. The increased SF may reflect normal procoagulant functions of placental trophoblasts (Galanakis et al. 1996). Our results also suggest that fibers bound to RBCs can explain the long known “red areas” of pathologic thrombi (Lippi and Favaloro 2018), as well as the “stable” RBC aggregates in capillaries of patients with various disorders (Brust et al. 2014; Glacet-Bernard et al. 1990; Harrison 2015). Additionally, there are a number manifestations of increased SF and RBC aggregation in the clinical laboratory. One comprises large clots that form in blood stored at 4 °C and trapped by the 170 μm or larger average pore transfusion filters through which all blood components are routinely infused to patients. Because the filters progressively clog and slow the infusion, they are typically changed every third infused RBC unit. When examined, filter trapped clots are long known to be laden with RBCs suggesting their binding to clot fibers. The second likely SF effect is interference in the agglutination tests routinely used for typing and antibody identification in blood banks particularly since blood banks currently use plasma for transfusion testing. Such tests are often hampered by unexplained and immunologically non-specific RBC rouleaux agglutinates and routine laboratory procedures exist to address or circumvent the problem (Cohn et al. 2020). A third SF effect may be encountered in routine blood smears of clinical specimens showing some rouleaux aggregates at the periphery or dense area of the smear and these are usually ignored and less dense smear areas are used to assess RBC morphology. These rouleaux agglutinates likely reflect low or normal levels of SF likely generated by phlebotomy. A fourth SF attribute in the clinical laboratory is its abundant presence in plasma and cryoprecipitate where its hypercoagulability (vide supra) likely results in enhanced hemostasis when infused during surgical procedures or for fg replacement. Evidence from a large clinical study tempts the suggestion that SF hypercoagulability plays a role in the venous thromboembolism associated with transfusion (Goel et al. 2018). That is, this study demonstrated an association of venous thromboembolism with perioperative transfusions in 0.9% of 47,410 patients from 525 teaching and non-teaching hospitals. The increase in the number of transfusions during a single hospitalization, moreover, was accompanied by a corresponding increase in venous thromboembolism.
