# Correction to: Novel characteristics of soluble fibrin: hypercoagulability and acceleration of blood sedimentation rate mediated by its generation of erythrocyte‑linked fibers

**DOI:** 10.1007/s00441-022-03618-9

**Published:** 2022-04-04

**Authors:** Dennis K. Galanakis, Anna Protopopova, Kao Li, Yingjie Yu, Tahmeena Ahmed, Lisa Senzel, Ryan Heslin, Mohamed Gouda, Jaseung Koo, John Weisel, Marilyn Manco‑Johnson, Miriam Rafailovich

**Affiliations:** 1 Dept. of Pathology, Stony Brook University School of Medicine, Stony Brook, NY USA; 2 Dept. of Cell and Developmental Biology, Univ. of Pennsylvania School of Medicine, Philadelphia, PA USA; 3 Dept. of Materials Science and Engineering, Stony Brook University, Stony Brook, NY USA; 4 Dept. of Medicine, Stony Brook University School of Medicine, Stony Brook, NY USA; 5 Dept. of Physiology and Biophysics, Stony Brook University, Stony Brook, NY USA; 6 Heme/Onc and Bone Marrow Transplantation, Children’s Hospital, Univ. of Colorado, Aurora, CO USA

## Correction to: Cell and Tissue Research https://doi.org/10.1007/s00441-022-03599-9

The authors regret that the last name of Dr. Lisa Senzel was captured incorrectly in the original article. It is now corrected in this erratum article.

The original article has been corrected.

